# Prothrombotic Fibrin Clot Phenotype in Patients with Deep Vein Thrombosis and Pulmonary Embolism: A New Risk Factor for Recurrence

**DOI:** 10.1155/2017/8196256

**Published:** 2017-06-27

**Authors:** Anetta Undas

**Affiliations:** Institute of Cardiology, Jagiellonian University School of Medicine and The John Paul II Hospital, Krakow, Poland

## Abstract

Prothrombotic fibrin clot phenotype, involving faster formation of dense meshwork composed of thinner and highly branched fibers that are relatively resistant to plasmin-induced lysis, has been reported in patients with not only myocardial infarction or stroke, but also venous thromboembolism (VTE), encompassing deep vein thrombosis (DVT), and/or pulmonary embolism (PE). Prothrombotic fibrin clot phenotype, in particular prolonged clot lysis time, is considered a novel risk factor for VTE as well as venous thrombosis at unusual location, for example, cerebral sinus venous thrombosis, retinal vein obstruction, and Budd-Chiari syndrome. Growing evidence from observational studies indicates that abnormal fibrin clot properties can predict recurrent DVT and PE and they are involved in serious complications of VTE, for example, thromboembolic pulmonary hypertension and postthrombotic syndrome. The purpose of this article is to review our current understanding of the role of fibrin clot structure and function in venous thrombosis with emphasis on clinical issues ranging from prognosis to therapy.

## 1. Introduction

Fibrinogen is converted to fibrin by thrombin [1, 2]. Thrombin-mediated release of fibrinopeptide A (FPA) and FPB from the N-termini of the A*α*- and B*β*-chains, respectively, results in the formation of fibrin monomer (*α*, *β*, *γ*)_2_. At the first stage of polymerization of fibrin monomers half-staggered and double-stranded protofibrils are formed supported by “A:a” interactions and then there is the assembly of protofibrils into fibers that are composed of thousands of protofibrils arranged side-by-side. Lateral aggregation is associated with FPB release and is likely caused by release of the C-termini of the A*α*-chains which interact and form bridges between protofibrils. Lateral aggregation is promoted mainly not only by intermolecular *α*C:*α*C interactions between protofibrils, but also probably by interactions between both *α*- and *γ*-chains [1, 2].

Formation of covalent cross-links formed by activated factor (F)XIII improves elasticity of fibrin clots and resistance to enzymatic degradation [3]. Dissolution of a fibrin clot is mediated by the interaction of tissue plasminogen activator (tPA) and plasminogen on fibrin fibers, which enhances the conversion of plasminogen to plasmin by tPA. Plasmin cleaves Lys-X and Arg-X bonds in the fibrin molecule. Plasminogen and tPA bind to lysine residues exposed by proteolysis of fibrin by plasmin. Plasmin that is bound to fibrin is relatively protected from inhibition by *α*_2_-antiplasmin bound to fibrin by FXIIIa. A key inhibitor of tPA is plasminogen activator inhibitor-1 (PAI-1). Additional fibrinolysis inhibitor, thrombin activatable fibrinolysis inhibitor (TAFI), downregulates plasminogen activation by removing plasmin-binding C-terminal lysine and arginine residues on fibrin [3].

Fibrin structure itself directly affects the rate of fibrinolysis, reflected by faster lysis of looser fiber networks regardless of the fiber thickness, which might affect the thrombotic risk [3, 6]. This effect of fibrin structure on lysis is mediated both by differences in accessibility of the clot to fibrinolytic proteins and differences in the binding of tPA and plasminogen to clots with different structures [7].

## 2. Evaluation of Fibrin Clot Characteristics

Architecture of a fibrin clot can be characterized by (1) the pore size typically estimated using clot permeability or permeation (Darcy's constant, or *K*_*s*_), a measure of clot surface allowing flow through networks under different hydrostatic pressures; (2) the lag phase by turbidimetry that reflects the time to the start of lateral protofibril aggregation; (3) maximum absorbance of a clot that reflects an average fibrin fiber thickness. Clot turbidity is related to the number of fibrin fibers, their thickness, the number of branching points, and the uniformity of fiber distribution [8–10]. The size of the pores in the fiber network or the density of clots can be assessed visually on scanning electron microscopy (SEM) or confocal microscopy images. The stiffness of fibrin clots generated from purified fibrinogen, plasma, or whole blood is evaluated using rheometry and its measure is the maximum elastic storage modulus. Other parameters of the clot including the fractal dimension reflecting its structural complexity can also be calculated using this approach usually in whole blood clots [11].

Since more than 30 years it has been proven that fibrin networks composed of thinner, highly branched fibers usually are less permeable, more rigid, and less susceptible to lysis [10, 12, 13]. The so-called prothrombotic fibrin clot phenotype involves faster formation of dense meshwork (reflected by lower *K*_*s*_ values) composed of thinner and highly branched fibers that are relatively resistant to plasmin-induced lysis. Contrary, fibrin networks composed of thick fibers have larger pores, which results in functional assays to increased permeability and susceptibility to fibrinolysis [10, 12, 13].

Nowadays the most commonly used assay to assess efficiency of fibrinolysis in patients is the measurement of clot lysis time (CLT) developed by Lisman et al. [14] in 2001. In this assay blood clotting is triggered by tissue factor (TF) in the presence of phospholipid vesicles and fibrinolysis is activated by addition of recombinant tPA together with TF [12]. Other assays to test fibrinolytic capacity use varying tPA concentrations with or without addition of thrombin before or after fibrin gel formation.

Fibrin clot properties can be studied when thrombin or other clotting activators are added to purified or recombinant fibrinogens. However, assays in which clots are generated from citrated plasma represent the most commonly used approach in large groups of patients. Structural and functional differences between plasma fibrin clots and those made from purified fibrinogen have been well documented. For example, fibrin network formed from citrated plasma is composed of thicker fibers making looser meshwork compared with that formed from purified fibrinogen [10, 12].

In summary, plasma fibrin clot permeability and clot lysis are valuable measures of clot structure and function, which can be used in evaluation of various diseases. However, these methods are not standardized and large interlaboratory differences are observed.

## 3. Modifiers of Fibrin Clot Properties

**Figure 1:**
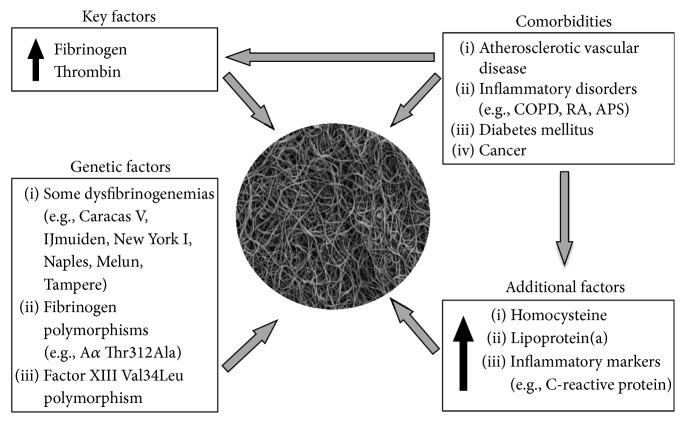
Factors that modify normal plasma fibrin clot phenotype to prothrombotic phenotype. A plasma fibrinogen concentration is a major determinant of clot properties. The larger fibrinogen concentrations, the denser fibrin networks. Alterations to fibrinogen function could be genetically determined and acquired largely associated with posttranslational modifications, for example, glycation or homocysteinylation. Environmental factors have a larger impact on clot phenotype and lysability, largely related to enhanced inflammation and thrombin generation observed in several common chronic diseases, including cancer and rheumatic disorders that represent well-established risk factors for venous thrombosis. COPD, chronic obstructive pulmonary disease; RA, rheumatoid arthritis; APS, antiphospholipid syndrome.

Experimental and clinical studies have identified a number of modifiers of clot structure, both genetically and/or environmentally determined, that in most cases are implicated—in a direct or indirect manner—in the occurrence of the prothrombotic fibrin clot phenotype (Figure 1).

Most of these factors are associated with increased risk of venous thrombosis. A key regulator of fibrin structure is the concentration of fibrinogen and its function. Variation in fibrinogen concentrations explains only up to 18% of the variation in clot permeability [15]. Other recognized modulators of fibrin structure involve acute phase proteins including C-reactive protein which rises together with fibrinogen in several common diseases associated with elevated risk of thrombosis (Figure 1).

It has been established that genetic factors explain 10–50% of variance in fibrin clot measures [16]. About 25% of rare congenital dysfibrinogenemias resulting from mutations in all three fibrinogen genes, despite low levels of functional fibrinogen measured using the Clauss assay, are linked with VTE and at least some of them are known to significantly alter fibrin clot structure [17]. The incomplete penetrance of the thrombotic phenotype in some dysfibrinogenemic subjects with the same genotype is highly suggestive of other genetic or environmental confounders. A stronger impact on fibrin clot characteristics could be related to common genetic polymorphisms largely in genes encoding three fibrinogen chains. A common *β*-chain polymorphism, due to a substitution of Lys with Arg at residue 448, has been shown to affect the clot structure [18], but not all studies demonstrated it [19]. A*α* fibrinogen Thr312Ala polymorphism has been reported to increase FXIIIa-catalyzed reactions and to lead to the formation of thicker fibrin fibers [20]. A functional splice variant of the *γ*-chain, fibrinogen *γ*′, with 20 new residues that contain binding sites for both thrombin and the FXIII B-subunit is also linked with an unfavorable prothrombotic clot phenotype [21]. Finally, FXIII Val34Leu significantly alters fibrin structure resulting in the formation of a clot with smaller pores and thinner fibers [22]. At high plasma fibrinogen concentrations, the FXIII Leu34 variant showed a protective effect through the formation of more permeable fibrin clots that are more susceptible to lysis, while at low fibrinogen concentration the effect was reversed, suggesting that protection against thrombosis by FXIII 34Leu only occurs in hyperfibrinogenemia [23]. Taken together, a role of common genetic factors in fibrin properties appears to be minor as compared to a broad spectrum of potent acquired factors largely driven by their complex proinflammatory and prothrombotic effects.

Wolberg et al. have shown that fibrin fiber mass-to-length ratio decreases with increasing prothrombin levels (above 100% of plasma levels), in a dose-dependent manner [24]. In both purified fibrinogen and plasma-based systems, clots produced in the presence of high thrombin concentrations are characterized by thin fibers that form a network with small pores [25].

Lipoprotein(a) that contains apolipoprotein(a), apo(a), with domains homologous with the kringle domains IV and V of plasminogen, has been found to be associated with lower clot permeability composed of thinner fibers and impaired susceptibility to fibrinolysis [26]. Small apo(a) isoforms are associated with abnormal clot characteristics and hypofibrinolysis [26].

A number of common diseases, including those known as the risk factors for thrombosis, have been demonstrated to deteriorate plasma fibrin clot phenotype by rendering them denser and resistant to lysis (Figure 1). Mechanisms underlying these associations are the consequence of posttranslational modifications such as oxidation, glycation, and homocysteinylation (often combined like in diabetes) and binding proteins to the fibrin fibers that can confound physiological interactions, for instance, with proteins involved in fibrinolysis [27–29]. The most common diseases related to thrombosis are inflammatory by nature. Reduced clot permeability and susceptibility to lysis have been observed in rheumatoid arthritis [30], chronic obstructive pulmonary disease [31], and inflammatory bowel disease [32]. Binding of C-reactive protein (CRP) with fibrinogen and fibrin [33] is likely to contribute to the prothrombotic clot phenotype as evidenced by correlations of CRP with plasma clot permeability and lysis [30–32].

## 4. Fibrin Clot Properties Assessed In Vitro in Patients with VTE

Venous thromboembolism (VTE), including deep vein thrombosis (DVT) and pulmonary embolism (PE), affects 1 to 3 per 1000 persons per year [34–36], which renders this entity a common medical condition associated with significant morbidity and mortality [37].

Growing evidence from multiple studies using various plasma-based assays indicates that both DVT and PE are associated with altered fibrin clot properties including impaired fibrinolytic capacity [10, 12, 38, 39]. The most compelling evidence for involvement of plasma fibrin clot characteristics in the pathogenesis of VTE is indirect and comes from several studies exploring efficiency of clot fibrinolysis. Impaired clot lysability is in part related to abnormal clot structure, which is commonly reflected by an inverse association between *K*_*s*_ and CLT reported in many disease states [10, 12]. Curnow et al. [40] showed that hypercoagulable patients with arterial thrombosis or VTE, pregnancy complications, or autoimmune diseases have increased fibrin generation and reduced fibrinolysis. Hypofibrinolysis measured in plasma using CLT values introduced by Lisman et al. [14] has been shown in subjects following the first DVT episode [41]. An increase in risk for DVT in patients with hypofibrinolysis and a 2-fold increased risk of DVT in subjects with CLT above the 90th percentile have been observed [41]. 77% of variation in CLT in venous thrombosis patients can be attributed to levels of PAI-1, TAFI, prothrombin, and *α*_2_-antiplasmin, but not to plasma fibrinogen [42].

Analysis of VTE patients demonstrated that three established risk factors for VTE, namely, oral contraceptives, immobilization, and the presence of FV Leiden, markedly increase the risk associated with the longest CLT; hypofibrinolysis with these factors gives 20-, 10.3-, and 8.1-fold increases in the VTE risk, respectively, compared with individuals with the shortest lysis time without the risk factors [43].

In 2009 unfavorably altered plasma fibrin clot properties were documented in patients with prior unprovoked VTE [44]. After excluding known thrombophilia, cancer, trauma, surgery, pregnancy, and other established risk factors, VTE patients have been found to have lower plasma clot permeability, higher maximum clot absorbance, and prolonged clot lysis than controls free of thrombotic events, while similarly unfavorable abnormalities were in relatives of VTE patients, which indicates genetic background of abnormal fibrin clot properties in VTE with no history of thrombotic events [44]. This study might suggest that abnormal plasma fibrin characteristics represent novel risk factors for idiopathic VTE. However, long-term cohort studies are needed to validate this hypothesis. Higher maximum clot absorbance usually inversely correlated with clot permeability is considered a marker of thicker fibers [8]. However in contrast to many in vitro studies in which clots composed of thinner fibers are more resistant to fibrinolysis, plasma clots from real patients with thrombotic manifestations can be composed of fibers with a similar or larger thickness as compared to the control subjects, but they are less permeable and poorly lysable. Patients following VTE represent such individuals, which suggests that plasma clots possess a far more complex structure in terms of its functional consequences.

Traby et al. [45] have reported that there is a weak association between CLT and risk of VTE recurrence only in women, who experienced a first unprovoked VTE without cancer or thrombophilia and were followed for an average of 46 months after anticoagulation withdrawal.

Using a global assay of fibrinolysis that tested the patient's blood fibrinolytic capacity by application of the euglobulin fraction of plasma to a preformed clot of plasminogen-rich bovine fibrin, Skov et al. [46] demonstrated that fibrinolytic capacity is impaired in VTE patients below 50 years compared with young stroke survivors and this difference remained significant after adjustment for multiple confounders including PAI-1.

A history of provoked and unprovoked DVT episodes has been later shown to be associated with unfavorable fibrin plasma clot properties [47]. It remains to be established whether unfavorable plasma fibrin clot phenotype may predispose to provoked DVT.

We have demonstrated that residual vein obstruction following DVT is linked with faster formation of denser plasma fibrin clots displaying impaired lysability [48]. These patients had 14% lower clot permeability and 11% longer lysis time, with no differences related to thrombophilia and duration or stability of anticoagulant therapy [48].

**Figure 2:**
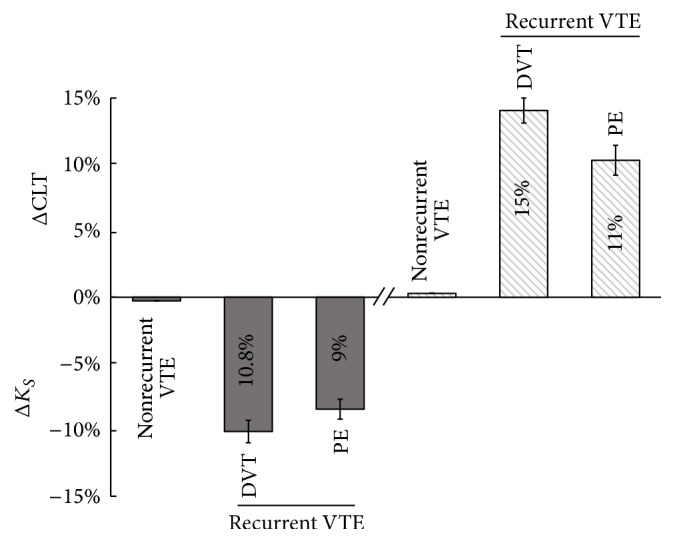
Recurrent VTE episodes during follow-up in relation to plasma fibrin clot properties measured after 3–6 months since the index event (based on [4, 5]). Lower clot permeability and prolonged clot lysis have been reported in patients who experienced VTE recurrences after anticoagulation withdrawal, with a larger impact on deep vein thrombosis. Data are shown as difference between means and confidence interval ranges in %. VTE, venous thromboembolism; DVT, deep vein thrombosis; PE, pulmonary embolism; *K*_*s*_, fibrin clot permeability coefficient; CLT, fibrin clot lysis time.

It remains unclear whether plasma fibrin clot features could predict recurrent VTE episodes. Regarding fibrinolytic parameters, in a two-centre case-control study performed in English and Dutch patients, hypofibrinolysis, defined as CLT values (at 3 months after discontinuation of the anticoagulant therapy) above the 90th percentile calculated in control subjects (>122 min), was associated with a 1.8-fold increased risk of a first VTE, while in the follow-up study the relationship between a recurrent VTE and hypofibrinolysis was estimated as weak (hazard ratio 1.5, *p* > 0.05) [49]. A prognostic value of plasma fibrin clot phenotype following DVT alone has been shown recently [50]. We have observed that subjects with recurrent DVT during follow-up were characterized by slightly lower plasma clot permeability and 15% longer CLT measured at 3 months since the index event compared with the remainder (Figure 2) [50].

Regarding PE, Martinez et al. have reported that clots made from plasma of patients with acute isolated PE were characterized by faster CLT and lower fiber density when compared with DVT alone [4]. Absent perfusion in some segments of the pulmonary arteries (perfusion defects) following PE detected on angiography has been shown to be associated with impaired fibrinolysis [51].

We have demonstrated recently the association of plasma clot properties and the risk of PE recurrence during a 4-year follow-up [52]. These associations were observed in subjects who discontinued anticoagulant therapy after the first episode of provoked or unprovoked PE (Figure 2). It is possible that screening for the prothrombotic plasma fibrin clot phenotype together with plasma D-dimer and thrombin generation may help identify patients at high risk of recurrent PE.

Analysis of thrombotic material removed from the right atrium and pulmonary (lobar and segmental) arteries of a patient with acute severe PE who underwent surgical embolectomy showed that distally located thrombi contain more densely packed fibrin fibers compared with the proximal thrombi from the lobar pulmonary arteries and the right atrial thrombus [5]. It indicates that abnormal fibrin is present in thrombi retrieved from patients with DVT and the subsequent PE.

In summary, DVT and/or PE are associated with the prothrombotic plasma fibrin clot phenotype especially reduced clot permeability and lysability, and these features might have a prognostic value both in the prediction of first and recurrent episodes.

## 5. Venous Thrombosis at Unusual Location

Studies on clot properties in patients who developed thrombosis at unusual location suggest that prothrombotic plasma clot phenotype contributes to such thromboembolic events, indicating that venous thrombosis is associated with certain common fibrin characteristics and the location of the event is largely driven by anatomical peculiarities or abnormalities.

The Budd-Chiari syndrome is a rare disorder caused by obstruction of the hepatic outflow tract, and its classic form involves thrombosis of one of the hepatic veins. This disease has been shown to be linked to less efficient fibrinolysis in part associated with elevated PAI-1 activity, but not with TAFI [53]. Marked differences in clot lysis between Chinese and European patients with this syndrome have been reported [54]. Data on clot architecture in this syndrome have not been published, but based on a significant inverse association of CLT with plasma clot permeability observed in most studies, it might expected that denser fibrin networks assessed in vitro are formed at least in some patients with this disease.

Cerebral sinus venous thrombosis (CSVT) is a rare disorder. Its recurrent episodes have been observed more frequently in patients with unfavorable clot phenotype; CVST was associated with 21% higher baseline fibrinogen, 20% lower plasma clot permeability and 17% greater fibrin mass within a clot [55]. Altered fibrin clot properties similar to those found following CVST have also been reported in patients who experienced retinal vein occlusion [56]. Prolonged lysis time associated with increased endogenous thrombotic potential following retinal vein thrombosis has been confirmed by Italian investigators [57].

In summary, prior thrombosis at unusual location may be linked to the prothrombotic plasma fibrin clot phenotype.

## 6. Inherited and Acquired Thrombophilia

It is estimated that genetic factors are responsible for up to 25% of unprovoked VTE [58]. Factor V Leiden is the most common inherited thrombophilia that occurs in 5% of Europeans. A heterozygous form of FV Leiden, perceived as a mild thrombophilic defect, has been reported to be associated with impaired efficiency of lysis in apparently healthy women below 50 years, which is independently predicted by the TAFI activity, and it has no significant effect on the fibrin network structure, reflected by clot permeability [59]. The FV Leiden paradox, that is, a relatively lower PE risk compared with high DVT risk in carriers, has not been elucidated [60]. Until now no evident fibrin clot-related mechanisms have been reported as a potential explanation of this paradox.

The G20210A prothrombin mutation is the second most common inherited abnormality that is found in 4.6–8% of patients after VTE and in 0.7–2.6% of the general white population [58]. The G20210A mutation carriers have about 20–25% higher prothrombin levels than noncarriers. Analysis of 32 carriers versus 30 noncarriers showed impaired fibrinolysis reflected by 14.5% longer CLT but this difference disappeared after TAFI inhibition [61]. They also confirmed that addition of prothrombin to normal plasma to raise prothrombin level to 125% and 150% results in longer CLT [61]. Our data indicated that also clot permeability is reduced in the G20210A prothrombin mutation carriers (A. Undas, unpublished data).

Genetically determined deficiencies of natural anticoagulants, that is, antithrombin, protein C, and protein S, increase the risk of a first VTE by at least 10-fold and are observed in <0.5% of the general white population with the incidence up to 5% among subjects with unprovoked VTE [58]. It is unclear whether such abnormalities alter fibrin clot properties. Reduced clot permeability and lysability have been reported in a single patient with antithrombin deficiency with normalization of clot permeability upon addition of antithrombin to plasma [62].

Antiphospholipid syndrome (APS) is a systemic autoimmune disease associated with thrombotic complications, including VTE, in the setting of detectable antiphospholipid antibodies [63, 64]. APS patients were characterized by higher maximum plasma clot absorbency, lower clot permeability, shorter lag phase, prolonged clot lysis time, lower maximum rate, and higher maximum level of D-dimer released from clots, also after adjustment for fibrinogen, body mass index (BMI), and smoking status [65]. There were no differences in plasma fibrin clot characteristics between primary and secondary APS. Patients with “double” or “triple” antibody positivity had less permeable plasma clots compared with those with one positive antibody, with no difference in clot lysis. Patients who experienced PE formed plasma fibrin clots of higher permeability and lysability than those with DVT alone. Clots generated from plasma of APS patients who experienced stroke and/or myocardial infarction were less permeable and were lysed slower compared with those with VTE alone [65]. This study is the first to show that APS is associated with prothrombotic plasma fibrin clot phenotype, with worse characteristics in patients following arterial thrombosis. Low clot permeability in APS was confirmed by Vikerfors et al. [66] and moreover, prolonged fibrinolysis was observed in particular in APS patients with previous arterial thrombosis. A role of microparticles in abnormal clot structure in APS patients has also been suggested [66]. Prothrombotic plasma clot properties in young or middle aged APS subjects following thrombotic events have been shown to be linked to progression of atherosclerosis measured in carotid arteries, which highlights again associations between fibrin-mediated mechanisms observed in both atherosclerosis and thrombosis [67].

In summary, there is evidence that most inherited thrombophilias and APS are associated with unfavorable plasma fibrin clot properties, which might contribute to their thromboembolic clinical manifestations.

## 7. Complications of VTE

Chronic thromboembolic pulmonary hypertension (CTEPH) is a severe complication that occurs in 1–4% of patients following PE. It has been reported that fibrin clots made from fibrinogen purified from patients with CTEPH are in part resistant to plasmin-mediated lysis as compared to healthy individuals [68]. This observation suggests that structural or functional abnormalities in fibrinogen molecules and the subsequent fibrin properties may be involved in the development of CTEPH by prolonged fibrin clot presence within the pulmonary arteries and stimulation of remodeling of the thrombi into fibrotic intravascular material. Interestingly, analysis of purified fibrinogen and fibrinogen gene sequencing of patients with CTEPH showed a relatively high incidence of inherited dysfibrinogenemias in which abnormal fibrin clot structure and lysis were described [69]. Moreover, it has been reported that patients with these CTEPH-associated dysfibrinogenemias have low clot turbidity, decreased porosity, and fibrinolytic resistance, along with disorganized fibrin networks composed of thinner fibers and more extensive fiber branching [70]. Since abnormal clot architecture and fibrinolytic resistance may contribute to incomplete clot resolution in this form of CTEPH, clot properties may help identify patients at risk of this complication. Given increased therapeutic options in CTEPH and their benefits [71, 72], efficacy of the therapy can also be in part determined by fibrin clot characteristics.

The most common complication of DVT is the postthrombotic syndrome (PTS) observed in 20–50% of patients within the first 1-2 years after the index event [73]. Patients who developed PTS suffer from light pain, occasional swelling, and venous ectasia, but with time they complain of chronic pain, tough edema, skin induration, and leg ulcer, which decrease the quality of life. The pathophysiology of PTS is not fully understood, but most investigators highlight a key role of systemic inflammation with the subsequent tissue remodeling and fibrosis [74]. Recently we have demonstrated that prothrombotic plasma clot phenotype, in particular lower clot permeability determined 3 months since the index event, predisposes to PTS [50]. Severe PTS was associated with more unfavorable clot variables, including almost 20% lower plasma clot permeability [50]. The Villalta scale, a measure of the severity of PTS, showed associations with clot permeability and lysability. We provided evidence that denser plasma fibrin clot formation and their impaired lysis may allow identifying patients following DVT who are likely to develop PTS.

## 8. Antithrombotic Agents and Fibrin Clot Properties

Anticoagulation is the primary approach to therapy of VTE. Heparins are commonly used as the first-line therapy in acute VTE, especially in cancer-associated episodes [75]. The current guidelines recommend lifelong anticoagulation for patients after a second unprovoked VTE and in most subjects after first episodes [36]. Vitamin K antagonists (VKA) have been the standard of care in VTE for 60 years. Nonvitamin K oral antagonists (NOACs) have shown similar efficacy and improved safety profile when compared to warfarin in VTE and they are now used worldwide [36].

It is easy to foresee that all anticoagulant agents that decrease thrombin generation and consequently fibrin formation may improve plasma fibrin clot properties. Unfractionated and low-molecular-weight heparins have been shown to improve clot properties as evidenced by analysis of nanostructure of fibrin clots in the absence of antithrombin [76].

Warfarin administration when International Normalized Ratio is in the range of 2-3 increased plasma fibrin clot permeability by 28–50% compared with the control clots [77]. Similar increases in clot permeability can be observed at therapeutic plasma concentrations of fondaparinux and apixaban (by 58–76% and 36–53%, resp.) [78]. Looser clot structure has also been shown in the presence of argatroban, bivalirudin, lepirudin, and danaparoid [79].

Rivaroxaban can improve clot properties as evidenced by formation of clots composed of thicker fibrin fibers and larger pores with 2-fold increased clot permeability and 3-fold faster lysis compared with the control clots generated in the absence of rivaroxaban [80]. Similar improvement in the presence of rivaroxaban has been reported in whole blood clots [81]. Direct thrombin inhibitors have been demonstrated to increase clot susceptibility to lysis and this effect was in part mediated by TAFI [82]. Reduced thrombin formation and suppressed thrombin-mediated reactions during NOAC treatment appear to account for the formation of less compact and more lysable fibrin clots. Whether the extent of changes in fibrin clots contributes to bleeding risk in VTE remains to be explored.

Low-dose aspirin has also been found to increase clot permeability and susceptibility to lysis, as demonstrated in clots generated from human plasma, purified fibrinogen and in cellular system [83, 84]. Acetylation of fibrinogen molecule is considered a major mechanism underlying this effect [83]. It is unclear whether these actions of aspirin contribute to its clinical outcomes observed also in secondary prevention of VTE.

Statins, a potent cholesterol-lowering class of drugs producing multiple antithrombotic actions which is increasingly used for other indications, have also been reported to increase clot permeability and lysability with no association with reduced cholesterol levels [85, 86]. It has been postulated that modulation of fibrin clot structure and function represents an additional beneficial effect of this widely used medications that have been shown to reduce the risk of VTE [87].

In summary, VKA, NOAC, and other anticoagulant agents favorably alter plasma fibrin clot properties. Less pronounced beneficial fibrin-related effects produce statins and aspirin, and these effects can be detected in vitro. Clinical relevance of these effects is unknown.

## 9. Concluding Remarks

The so-called prothrombotic characteristics of a fibrin clot, including dense fiber networks displaying reduced plasmin-induced lysability measured in vitro in plasma, are considered a potential novel risk factor for VTE like MI or ischemic stroke. Observational studies have demonstrated that altered plasma clot phenotype, in particular low clot permeability and CLT, characterize patients following VTE and thrombosis at unusual sites as well as those who experienced recurrent VTE or PE during follow-up. The precise molecular mechanisms underlying this association are still poorly understood and involve genetic and acquired factors with a major contribution of increased thrombin generation and inflammation. Of note, plasma fibrin networks are favorably modulated in subjects receiving not only various anticoagulant agents, but also aspirin or statins, which might contribute to beneficial effects of these medications in terms of prevention of VTE.

It remains to be established to what extent unfavorable fibrin clot properties affect the risk of first or recurrent DVT and PE. Further large prospective studies are needed to evaluate a prognostic value of fibrin clot measures in a broad spectrum of venous thromboembolic disorders.
